# Supplementary material for: Analysis of PPARγ Signaling Activity in Psoriasis
Source: Int J Mol Sci. 2021 Aug 10;22(16):8603. doi: 10.3390/ijms22168603 (PMC8395241; doi:10.3390/ijms22168603)
Supplement: Supplementary file 1 [file ijms-22-08603-s001.zip › Supplemental materials_Analysis of PPARg signaling activity in psoriasis/Pathway models/Models images and html files/PPARG expressed in cells assotiated with psoriasis/236447.html]

inflammatory cell


# CellType inflammatory cell

|  |  |
| --- | --- |
| URN | urn:agi-ncimcelltype:C0440752 |
| Total Entities | 0 |
| Connectivity | 4235 |
| Name | inflammatory cell |

---

|  |  |
| --- | --- |
| Pathway | PPARG expressed in cells assotiated with psoriasis |
|  | Neighbors of inflammatory cell |

---

|  |  |
| --- | --- |
| MedScan ID | 10110625 |

---

|  |  |
| --- | --- |
| Alias | inflammatory cell |
|  | inflammatory cell reactions |
|  | inflammatory cell reaction |
|  | inflammatory cells |

---

|  |  |
| --- | --- |
| NCIm ID | C0440752 |

---
